# Restaurant Cooking Trends and Increased Risk for Campylobacter Infection

**DOI:** 10.3201/eid2207.151775

**Published:** 2016-07

**Authors:** Anna K. Jones, Dan Rigby, Michael Burton, Caroline Millman, Nicola J. Williams, Trevor R. Jones, Paul Wigley, Sarah J. O’Brien, Paul Cross, for the ENIGMA Consortium

**Affiliations:** Bangor University, Bangor, Wales, UK (A.K. Jones, P. Cross);; University of Manchester, Manchester, UK (D. Rigby, M. Burton, C. Millman);; University of Liverpool, Neston, UK (N.J. Williams, T.R. Jones, P. Wigley, S.J. O’Brien)

**Keywords:** food poisoning, Campylobacter jejuni, undercooked chicken, restaurant practices, cooking cultures, campylobacteriosis, bacteria

## Abstract

In the United Kingdom, outbreaks of *Campylobacter* infection are increasingly attributed to undercooked chicken livers, yet many recipes, including those of top chefs, advocate short cooking times and serving livers pink. During 2015, we studied preferences of chefs and the public in the United Kingdom and investigated the link between liver rareness and survival of* Campylobacter*. We used photographs to assess chefs’ ability to identify chicken livers meeting safe cooking guidelines. To investigate the microbiological safety of livers chefs preferred to serve, we modeled *Campylobacter* survival in infected chicken livers cooked to various temperatures. Most chefs correctly identified safely cooked livers but overestimated the public’s preference for rareness and thus preferred to serve them more rare. We estimated that 19%–52% of livers served commercially in the United Kingdom fail to reach 70°C and that predicted *Campylobacter* survival rates are 48%–98%. These findings indicate that cooking trends are linked to increasing *Campylobacter* infections.

## Abstract 2

Perceived consumer preferences for rare chicken liver are increasing risk for campylobacteriosis.

Foodborne illness is very costly, comprising medical expenses, loss of earnings, and reduced quality of life. In the United States, the annual healthcare cost is ≈$14 billion annually (*1*); in the United Kingdom, it is £1.8 billion (*2*). The foodborne illness most commonly responsible for these costs is campylobacteriosis (*3*–*5*). In the United States, cases increased by 13% between 2006–2008 and 2013 (*6*). In the United Kingdom, *Campylobacter* accounted for over half of the estimated 500,000 cases of foodborne disease during 2011–2012 (*3*,*7*); in the United States, it accounts for 9% of foodborne disease cases annually (*4*).

Foods implicated as *Campylobacter* vehicles include poultry, red meat, milk, and water (*7*–*11*). Studies of outbreaks and sporadic cases have identified the principal source of infection as undercooked chicken meat (*9*–*14*). In the United Kingdom, increasing numbers of outbreaks are attributed to undercooked chicken livers (*9*) despite the fact that the UK Food Standards Agency (FSA) has provided guidelines for safely cooking them. These increased infections seem to have coincided with a trend among leading chefs to advocate minimal cooking of chicken livers, despite recommendations to maintain liver cores at 70°C for 2–3 minutes to ensure they are *Campylobacter* free (*15*).

Although the association between consuming chicken livers and infection with *Campylobacter* is well known (*9*), the underlying reasons for the changing epidemiology of outbreaks associated with chicken liver consumption are unclear. We hypothesized that the trend toward including rarer, pinker meat in the recipes of leading chefs and by mass media representation of meat cooking may be contributing to changes in the way chicken livers are consumed.

We therefore conducted an interdisciplinary investigation by using a combination of methods from social and biological sciences. Participants were selected from the UK population, and the study was conducted during 2015. Our study objectives were 1) to investigate the ability of chefs and members of the public to identify cooked chicken livers that meet FSA guidelines for safe cooking, 2) to elicit the preferences of chefs and the public regarding the rareness of chicken livers, and 3) to model the survival of *Campylobacter* in chicken livers sautéed to various core temperatures.

## Methods

### Participants

We recruited a quota-based sample of 1,030 members of the UK public via an online market research panel (http://www.researchnow.com). Quotas were used to ensure representativeness in terms of age groups and social class. The quota permitted an unequal split by sex (up to 70% women) because in the United Kingdom, food preparation at home is more commonly performed by women than men. We also recruited 143 chefs through face-to-face convenience sampling at culinary shows and competitions and by online culinary forums.

All participants gave informed consent. Respondents were debriefed on the purpose of the survey after completion and given the opportunity to withdraw their data. Ethical approval was obtained from the College of Natural Science Ethics Committee at Bangor University (CNS/2014/AJ1).

### Preparation of Visual Aids

To prepare cooked chicken liver dishes to serve as visual aids, we used methods similar to those used in studies of hamburgers (*16*) and beefsteaks (*17*). A chef cooked 7 batches of chicken livers for various times, recorded the maximum core temperature for each batch, and arranged each batch on a plate for photography by a professional photographer. The process was repeated (without the temperature being recorded) for 3 other meats (duck breasts, lamb racks, and beef burgers).

### Surveys of Preference and Knowledge

**Figure 1:**
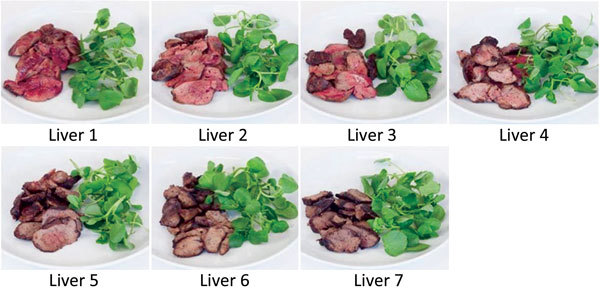
Chicken liver images, in order of cooking time/rareness, used in survey to determine preferences and knowledge of safe cooking practices among chefs and the public, United Kingdom.

To determine preferences and knowledge of safe cooking practices among chefs and members of the public, we used the images of cooked chicken livers as visual aids. The images were presented in surveys (online and print), arranged in order of cooking time/rareness (Figure 1). The surveys for chefs and the public were similar, except that the chefs were asked about serving preferences and the public was asked about eating preferences. 

To avoid biases (such as social desirability bias) resulting from respondents perceiving the survey to be about food safety, we described the survey as being about food preferences. Respondents were first asked preference questions about 3 of the 4 meats (in random order) to obscure the focus on chicken livers and safety. Chefs were asked to indicate which chicken liver dish was cooked “the way you would like to serve it” and “the way you think most customers would like it.” Members of the public were asked which dish they would prefer if “eating out” and “eating at home.”

Respondents were subsequently asked which chicken liver dish (if any) was the first they thought would meet FSA safe cooking guidelines. Additional questions were asked about perceived trends and influences regarding cooking meat, dining habits, and demographic information such as class and age. Chefs provided additional information about their current position, such as their training and industry experience.

### *Campylobacter* Survival

To prepare a suspension of *Campylobacter* for experimental inoculation, we streaked *Camplyobacter jejuni* M1 strain (sequence type 137, clonal complex 45) on Columbia agar base containing 5% defibrinated horse blood, incubated it at 37°C under microaerobic conditions for 48–72 h, and then inoculated it into *Camplyobacter* enrichment broth. After subculture for another 24 h, a bacterial suspension was prepared in maximum recovery diluent to an optical density of 600 nm (≈10^9^ CFU/mL). The culture broth was diluted in *Camplyobacter* enrichment broth to give a suspension of ≈10^5^ CFU/mL for inoculation into fresh chicken livers.

The fresh chicken livers were purchased in packs from supermarkets and sorted into batches of 4 with similar weights. The connective tissue was cut between the 2 liver lobes, with the weight of the larger lobe recorded and assigned for inoculation with *Campylobacter* broth suspension; 4 livers were assigned to each cooking batch. A 1-cm^2^ area of each liver was scored at its thickest point by using a sterile scalpel blade and injected with 100 μL (≈10^4^ CFU) of culture broth, corresponding to the highest levels of *Campylobacter* reported to be found in naturally contaminated livers (*18*).

For each cooking time, 10 g butter was heated in a frying pan over moderate to high heat on an electric cooktop; when the butter had finished frothing, the 4 inoculated liver lobes in the batch were added. The maximum core temperature of the largest and smallest liver in each batch was recorded. To determine the survival of the inoculated M1 strain of *C. jejuni* within the cooked livers, we placed each liver in a sterile petri dish and a 4–5-g portion around the scored inoculated region was removed and added to a Stomacher bag (Seward BA6040, Worthing, UK); 10 mL of Exeter broth was added to each bag before Stomaching (mechanical pounding of the outer surface of the bag to remove bacteria) for 1 min. The homogenized suspension was poured into a 20-mL universal container and incubated at 41°C under microaerobic conditions (Variable Atmosphere Incubator; Don Whitely Scientific, Shipley, UK) for 24 h, after which 1 loopful of broth was plated onto *Campylobacter* blood-free medium (modified charcoal cefoperazone deoxycholate agar, containing cefoperazone and amphotericin) at 41°C under microaerobic conditions for 48–72 h. We picked 1 typical *Campylobacter* colony from at least 1 plate in each batch and confirmed it as *C. jejuni* by PCR; for a cooked liver to be deemed positive, 1 isolate per batch was confirmed as *C. jejuni* positive (*19*).

### Data Analyses

**Figure 2:**
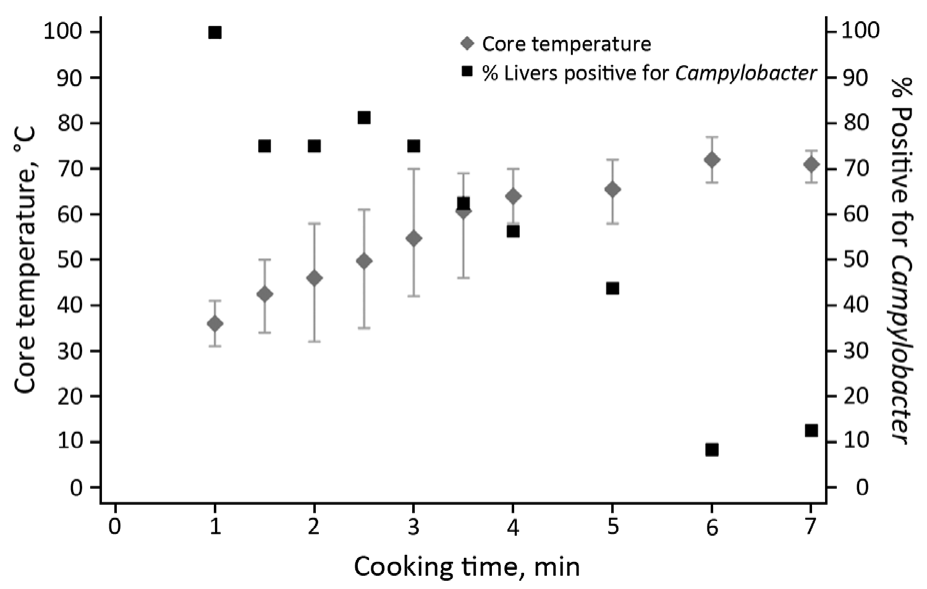
*Campylobacter* survival in cooked (pan-fried) chicken livers, by cooking time and temperature. Error bars represent minimum and maximum temperatures reached.

We modeled the probability of survival for the 60 livers for which temperature and *Campylobacter* presence/absence after cooking were recorded. We used logistic regression to model the relationship between the core temperature of the livers and the survival of C*ampylobacter*. The probability of *Campylobacter* survival as a function of core temperature was modeled via estimation of a logit model, which captured the nonlinear temperature-survival relationship (Figure 2). Parameter estimates were obtained by using logistic regression (Stata logit command; StataCorp LP, College Station, TX, USA) on the binary variable indicating Camplyobacter survival (1 = survival, 0 = nonsurvival) in a sample of 60 cooked chicken livers. Temperature was the maximum core temperature recorded for the batch from which the chicken liver was taken. This model was used to assign predicted survival rates for each photographed chicken liver dish.

We used the Kolmogorov Smirnov 2-sample test to compare differences in the distribution of knowledge and preferences between groups (chefs and the public). We investigated within-person differences by using the Wilcoxon signed-rank test for paired data. Ordered logit models (*20*) were estimated to determine the effects of observable characteristics on respondents’ preferences for chicken liver rareness and their choices of FSA-compliant livers.

## Results

### *Campylobacter* Survival

We discuss the results of the *Campylobacter* survival experiment first because an understanding of those results is useful for interpreting the preferences and knowledge analyses. The relationship between core temperature and *Campylobacter* survival rate was inverse (Table; Figure 2). Of the 32 batches of 4 inoculated livers, the shortest cooking time was 1 minute, leading to a mean core temperature of 36°C and a 100% *Campylobacter* survival rate. At the maximum mean core temperature (72°C), *Campylobacter* survival rate was 8.3%.

**Table:** *Campylobacter* survival in cooked chicken liver, by replicate*

Variable	Cooking time, min									
	1	1.5	2	2.5	3	3.5	4	5	6	7
Replicate 1										
No. positive	4.0	3.0	3.0	3.0	4.0	3.0	2.0	3.0	ND	ND
Mean weight, g	41.5	41.5	43.8	41.5	41.5	41.5	40.3	40.8	ND	ND
Mean core temp, °C	36.0	46.0	44.0	41.0	47.5	55.5	60.5	61.5	ND	ND
Replicate 2										
No. positive	ND	3.0	3.0	4.0	3.0	3.0	3.0	3.0	0	ND
Mean weight, g	ND	34.0	34.0	34.0	34.3	34.0	34.3	34.5	34.3	ND
Mean core temp, °C	ND	39.0	42.5	44.0	50.5	59.0	65.5	65.0	72.0	ND
Replicate 3										
No. positive	ND	ND	2.0	4.0	4.0	4.0	3.0	3.0	1.0	1.0
Mean weight, g	ND	ND	40.0	40.3	39.3	40.5	40.5	40.3	39.5	39.0
Mean core temp, °C	ND	ND	41.5	55.5	57.5	61.0	69.0	64.0	69.0	72.5
Replicate 4										
No. positive	ND	ND	4.0	2.0	1.0	0.0	1.0	1.0	0	0
Mean weight, g	ND	ND	25.8	26.3	28.0	26.5	27.3	24.8	27.8	29.5
Mean core temp, °C	ND	ND	56.0	58.5	63.5	67.5	61.0	71.5	75.0	69.5
No. livers	4	8	16	16	16	16	16	16	12	8
No. positive	4	6	12	13	12	10	9	10	1	1
Mean no. positive per batch of 4	4.0	3.0	3.0	3.3	3.0	2.5	2.3	2.5	0.3	0.5
Overall mean % of positives	100	75.0	75.0	81.3	75.0	62.5	56.3	62.5	8.3	12.5
Overall mean liver weigh, g	41.5	37.8	35.9	35.5	35.8	35.6	35.6	35.1	33.8	34.3
Overall mean core temperature, °C	36.0	42.5	46.0	49.8	54.8	60.8	64.0	65.5	72.0	71.0

*ND, not detected.

The logistic model predicted a survival rate of 98% in liver with core temperature that reached 52°C (liver 1) and equivalent survival rates of 95% and 48% at core temperatures of 56°C and 66°C (livers 2 and 3). Liver 4 reached a maximum temperature of 70°C, but the temperature was not held for the recommended 2 minutes; predicted *Campylobacter* survival rate was 22%. Livers 6 and 7 met the FSA guidelines, and their predicted *Campylobacter* survival rate was <0.001%.

### Preferences and Knowledge of the Public

Of the 1,030 members of the public surveyed, 43.0% ate chicken livers and hence were asked to select the chicken liver dishes they preferred and which they thought met FSA guidelines. Half (49.3%) of all male respondents and 38.4% of all female respondents ate chicken livers. Rates of chicken liver consumption varied by age group: 18–34 years, 34.7%; 35–44 years, 44.7%; 45–54 years, 49.0%, 55–64 years: 51.5%; and >65: 42.9%. Chicken livers were eaten by half (51.0%) of respondents belonging to UK socioeconomic grouping ABC1 (upper, middle, and lower middle class) and 32.3% of those belonging to C2DE (working class and those at the lowest level of subsistence).

**Figure 3:**
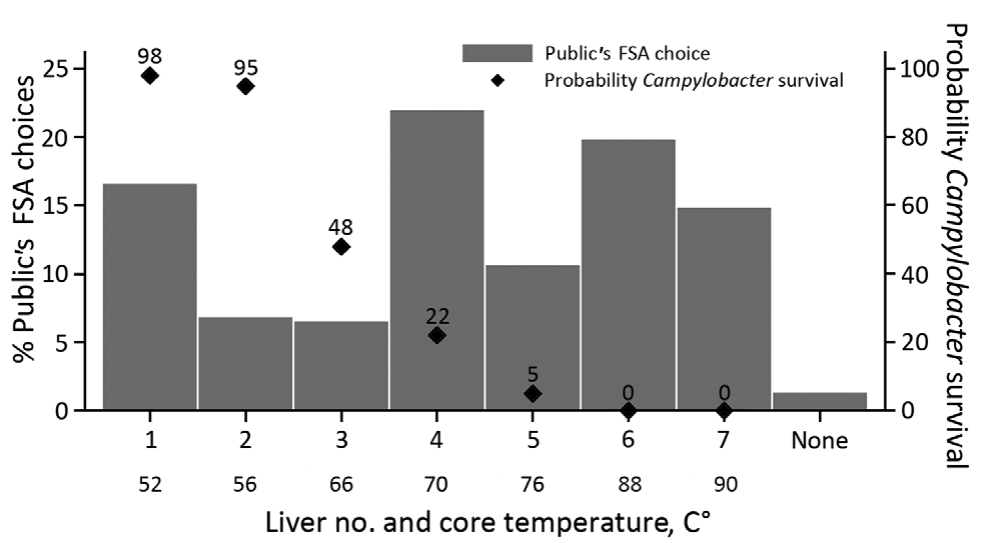
Rarest chicken livers visually identified by members of the public as complying with FSA cooking guidelines and associated core temperatures and probabilities of *Campylobacter* survival in survey to determine preferences and knowledge of safe cooking practices among chefs and the public, United Kingdom. Liver image numbers correspond to those shown in Figure 1. FSA, Food Standards Agency.

Members of the public poorly identified whether a chicken liver met FSA guidelines for safe cooking (Figure 3). Thirty percent identified livers 1–3 as being safe to eat; the predicted rates of *Campylobacter* survival in these livers were 48%–98%. Another 22% thought that liver 4 (*Campylobacter* survival rate 22%) was safe to eat.

**Figure 4:**
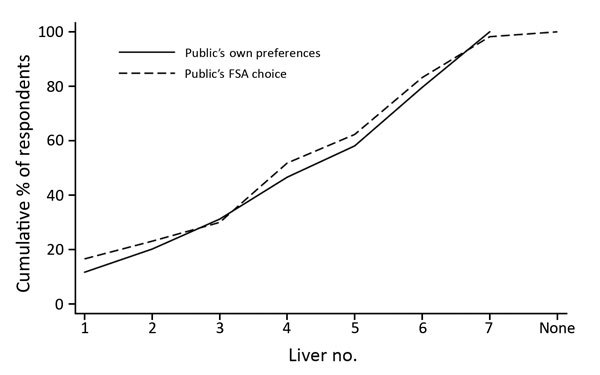
Proportion of public identifying which chicken liver dishes they preferred and which they believed complied with FSA cooking guidelines in survey to determine preferences and knowledge of safe cooking practices among chefs and the public, United Kingdom. Liver image numbers correspond to those shown in Figure 1. FSA, Food Standards Agency.

No significant difference was found between the public’s choices of FSA-compliant livers and their preferences when dining out (p = 0.776, Wilcoxon signed-rank test; n = 386) (Figure 4); respondents were consistent between what they wanted to eat and what they thought was safe. Respondents showed a significant preference for pinker livers when eating out rather than at home (p = 0.007, Wilcoxon signed-rank test; n = 446). Paradoxically, respondents reported being more concerned about food safety when eating out than at home (p<0.001, Wilcoxon signed-rank test; n = 999).

Ordered logit results (not reported) identified no systematic differences in rareness preferences by respondent sex, age, or class. Livers that were more pink were preferred by respondents who described themselves as adventurous (p<0.030, n = 444) and who were less concerned about restaurant food safety (p<0.001, n = 444).

### Perceptions and Knowledge of Chefs

Among the 143 chefs, of those who indicated their sex, 134 (88%) were male. Among the 141 who indicated their type of work, 31.9% worked in fine dining, 17% in contract catering, 11.3% in casual restaurants, 5.7% in pubs, and 19.1% in multiple kitchen types. The most commonly held position among 131 chefs who responded was head chef (54.0%), followed by chef trainer (11.5%), chef de partie (10.7%), commis chef (6.9%), and sous chef (6.1%).

**Figure 5:**
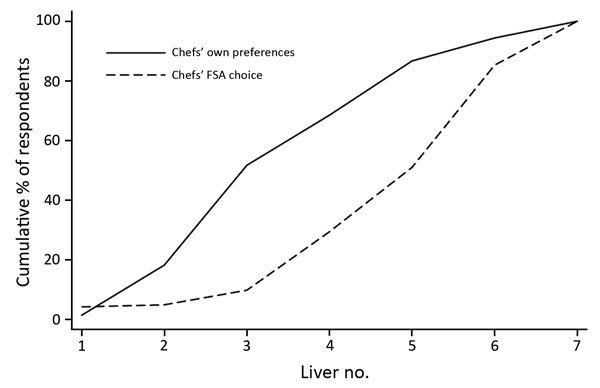
Proportion of chefs identifying which chicken liver dishes they preferred and which they believed complied with FSA cooking guidelines in survey to determine preferences and knowledge of safe cooking practices among chefs and the public, United Kingdom. Liver image numbers correspond to those shown in Figure 1. FSA, Food Standards Agency.

Chefs were much better than members of the public at identifying whether a chicken liver met FSA guidelines; only 9.8% of chefs (vs. 30% of the public) selected livers 1–3 as being FSA compliant (Figure 5), and another 19.8% thought that liver 4 met FSA guidelines. Although they outperformed the public, 30% of the chefs identified livers with *Campylobacter* survival rates of 22%–98% as being FSA compliant.

Chefs preferred to serve livers more pink than they thought would meet FSA guidelines (p<0.001, Wilcoxon signed-rank test; n = 143) (Figure 5). Chefs also preferred to serve livers substantially more pink than the public preferred when eating out (p<0.001, Kolmogorov-Smirnov 2-sample test). Chefs’ perceptions of customers’ preferences for rareness differed significantly from customer’s true preferences: not only did chefs prefer to serve livers more rare than customers wanted them served, they also thought that customers wanted chicken livers more rare than the customers themselves indicated (p = 0.008, Kolmogorov-Smirnov 2-sample test).

As with the members of the public, in the ordered logit model to explain serving preferences, chef preference for pinkness of served livers did not vary according to chef characteristics such as age, sex, and class. The only significant results indicated that chefs holding senior positions preferred to serve liver more pink than did their less experienced colleagues holding junior kitchen positions (p = 0.002).

### Culinary Trends

Almost half (47.8%) of the members of the public sampled agreed that “cooking programmes on TV and/or recipes in magazines have influenced the way the general public cook meat, people now serve it pinker in the middle.” Among chefs, >45% agreed that they had noticed a trend of rarer and pinker chicken livers on television, in recipes, and among other chefs.

## Discussion

Members of the public poorly identified whether chicken livers had been cooked to a safe microbiological state. Their preferences for chicken livers were consistent with their (often inaccurate) perceptions of safely cooked livers. Among chefs, these variables differed; chefs outperformed the public at identifying whether chicken livers had been cooked to FSA guidelines. We found that chef preferences for serving chicken livers were inconsistent with their perceptions of safe cooking—they preferred to serve livers more rare than is microbiologically safe and believed that their customers also prefer them more rare than is safe. Chefs systematically overpredicted their customers’ preferences for pinkness of livers served. This finding probably means that an estimated 19%–52% of livers being served in commercial UK food establishments fail to reach a core temperature of 70°C and could have *Campylobacter* survival rates of 48%–98%.

Chefs preferred rarer livers than the FSA guidelines would recommend. Chefs (correctly) thought that customers preferred livers less rare than their own preferences (p<0.001, Wilcoxon signed-rank test), but they still overestimated customers’ preference for pinkness. Chefs’ preferences, rather than their ignorance of FSA microbiological guidelines, seem to be leading them to serve undercooked livers. This finding resonates with previous findings that knowledge is not necessarily a driver of behavior (*21*–*23*). We contend that the explanation for the discrepancy between cooking practices and recommended guidelines is a cultural one, resulting in preferences for taste and texture overriding the desire to avoid foodborne illness (*24*–*26*). In extremis, this preference ultimately led chef Raymond Blanc to remove liver dishes from the menu rather than increase cooking times/temperatures after cases of campylobacteriosis were attributed to diners having eaten liver in his restaurant (*27*).

**Figure 6:**
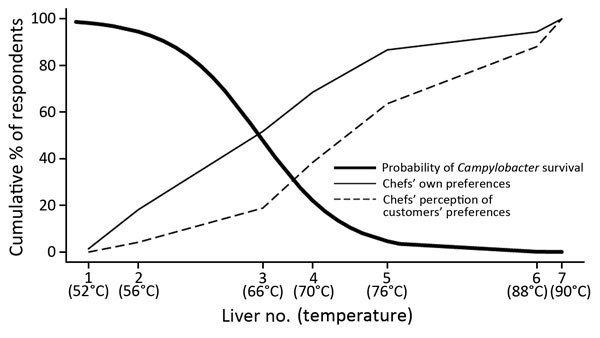
Proportion of chefs identifying which chicken liver dishes they preferred and which they believed their customers would prefer and associated probabilities of *Campylobacter* survival in survey to determine preferences and knowledge of safe cooking practices among chefs and the public, United Kingdom. Liver image numbers correspond to those shown in Figure 1.

The public health implications of the contrast between chef preferences and safe practices depend largely on what chefs provide for customers. Given that chefs prefer livers more pink than they believe customers do, we take the chef perception of customer preference as the lower bound and chefs’ own preference as the upper bound on the rareness of chicken livers served. This finding implies that 18.9%–51.7% of livers being served in commercial UK food establishments are failing to reach a core temperature of 70°C and have *Campylobacter* survival rates of 48%–98% (Figure 6). Extending the range of livers considered unsafe to liver 4 from our testing implies that 38.5%–68.5% of chicken livers being served commercially may have *Campylobacter* survival rates of 22%–98%.

This preference for rare chicken livers is part of a broader shift in contemporary cooking culture toward rarer meats, a trend that is reflected in the mass media (*28*,*29*) but not yet in the peer-reviewed literature. Periodically, the preference among chefs for serving rarer meat results in conflicts with recommendations of public health officials (*30*–*32*). The trend toward serving meat more pink has now extended from meats such as beefsteaks to meats such as chicken livers, for which the microbiological risks associated with rareness are far greater.

Our interdisciplinary approach, using relatively large samples of chefs and members of the general public, provides a unique insight into the possible public health implications of a divergence between preferences and safe cooking. A limitation of our approach is basing selection of preferred dishes on visual inspection alone. However, an experimental design that enabled respondents to physically assess cooked dishes would have severely limited study size. Another limitation is use of a laboratory-cultured inoculum, which might be less heat resistant than naturally occurring bacteria. Therefore, the projected death rates might be overestimated, and undercooked livers might pose even more of a risk than this study suggests. Our results relate to the *C.* *jejuni* M1 strain only; other *Campylobacter* strains may exhibit different survival characteristics. *Campylobacter* survival is reported here in terms of presence or absence, not as colony counts. Results indicate public risk for exposure to *Campylobacter*, not risk for infection or subsequent illness. The low doses required for infection and illness (*33*,*34*) are part of a stochastic process that can happen at any dose, suggesting that the presence of any *Campylobacter* in cooked livers poses a public health threat.

Because all experimental livers were inoculated with *Campylobacter*, our results have been framed in terms of probability of *Campylobacter* survival rather than exposure. Hence, our reported rates at which chefs serve *Camplyobacter*-positive livers may be slightly overestimated.

The temperature–survival results presented here, supported by those of Whyte et al. (*15*), suggest that the chicken liver cooking techniques practiced by many chefs, and promoted in the culinary and mass media, are leading to increased exposure to *Campylobacter*. The role of celebrity chefs and the mass media in pushing the trend toward serving pink meat were evident in our results. Recipes by top chefs frequently recommend serving chicken livers pink in the middle in warm salads, pâtés, and parfaits (*35*,*36*). This trend toward pink resonates with our estimate, based on our survey and experimental results, that 19%–52% of livers served in UK food outlets do not reach a core temperature of 70°C and our predicted *Campylobacter* survival rates of 48%–98%. Given *Campylobacter* prevalence rates among UK retail chicken livers (81%–100% externally, 90% internally [*15*,*37*]), our results suggest that contemporary cooking trends are leading to the “gourmet-fication” of foodborne disease.
